# Viral Infections in Kidney Transplant Recipients: Current Practice and Updates

**DOI:** 10.3390/jcm15031166

**Published:** 2026-02-02

**Authors:** Kayinsola Kehinde Babatunde, Donnchadh Reidy, Dearbhail Ni Cathain, Sam Kant

**Affiliations:** Department of Nephrology, St. Vincent’s University Hospital, D04 T6F4 Dublin, Ireland; babatundekehinde10@outlook.com (K.K.B.); donnchadhreidy@svhg.ie (D.R.);

**Keywords:** kidney transplantation, viral infections, immunosuppression

## Abstract

Kidney transplantation is considered the gold standard treatment for patients with end-stage kidney disease. Historically, outcomes in kidney transplantation have been focused on reducing rates of rejection as the dominant cause of graft loss. However, managing the risk of rejection with infection continues to be a delicate balancing act for transplant physicians. It has long been recognised that viruses are an important cause of morbidity and mortality in immunosuppressed patients with significant implications for kidney graft function and patient outcomes worldwide. This is a review article with literature selected from the PubMed database using relevant terms related to kidney transplantation and infectious diseases. This article focuses on the key viruses affecting kidney transplant recipients, including cytomegalovirus, polyoma virus, Epstein–Barr virus, varicella zoster virus, adenovirus, hepatitis B and C, and new emerging viruses. It examines differing epidemiology, diagnostic challenges, screening methods, and antiviral treatments. Key challenges for the international nephrology community include increased global mobility resulting in rapid shifts in viral epidemiology, increasing antimicrobial resistance, virus-associated malignancies, and suboptimal assays for screening donors and transplant recipients.

## 1. Introduction

Kidney transplantation has greatly improved outcomes for patients with end-stage kidney disease. With increased survival, it is now recognized that viral infections are a leading cause of post-transplant complications such as morbidity, mortality, and allograft dysfunction in this immunosuppressed group. The infection risk depends on the immunosuppression level, donor–recipient serostatus, and prior exposure. Transplant nephrologists must balance immunosuppression to prevent rejection with limiting infections that can lead to allograft dysfunction, promote rejection, cancer, and cardiovascular issues [1,2].

This review synthesizes the recent evidence and international guidance on the epidemiology, pathogenesis, risk stratification, diagnostics, prevention, and management of major viral infections in kidney transplantation, focusing on the clinical nephrology practice.

## 2. Cytomegalovirus

### 2.1. Introduction

Human cytomegalovirus (CMV) is a widespread double-stranded DNA virus classified within the Betaherpesvirinae subfamily. CMV infection is generally acquired during childhood or adolescence and exhibits a seroprevalence ranging from 30% to 90%, depending on geographic location and age. After primary infection, CMV remains latent and opportunistic, with the capacity to reactivate in immunocompromised individuals, such as kidney transplant recipients. CMV constitutes a significant pathogen in the context of kidney transplantation due to its associated morbidity and mortality, which result from both its direct and indirect effects on transplant outcomes [1,2].

In 1921, Goodpasture and Talbot proposed that a viral agent was responsible for the ‘cytomegalia’ phenomenon observed in CMV infection, as opposed to the initially suspected parasitic infection. Weller and colleagues designated the virus as cytomegalovirus to reflect its capacity to induce cellular enlargement [3,4]. Cytomegalovirus (CMV) infection affects between 50% and 90% of kidney transplant recipients, depending on the serostatus combinations of donors and recipients [1,5]. The highest risk is identified in cases involving seropositive donors and seronegative recipients (D+/R−). Seropositive recipients (D−/R+) are classified as medium-risk, whereas seronegative donor–recipient pairs (D−/R−) are associated with the lowest risk [6].

### 2.2. Pathogenesis and Screening

Cytomegalovirus (CMV) replicates in various tissues, including the renal allograft, and triggers immune activation and modulation. The expression of immediate early genes is essential for CMV reactivation, often stimulated by tumour necrosis factor-alpha (TNF-α) and other inflammatory cytokines. CMV exhibits immunomodulatory properties that suppress natural killer (NK) and T cell responses, thereby facilitating viral persistence. Post-transplant immunosuppressive therapy affects cellular immunity, particularly impacting CD4+ and CD8+ T lymphocytes, which are responsible for limiting viral replication and thereby increasing the risk of CMV disease [1,2].

### 2.3. Risk Stratification and Screening

Monthly monitoring of cytomegalovirus (CMV) is advised during the initial six months following transplantation, with subsequent assessments being less frequent. Serological testing is predominantly used for risk stratification in pre-transplant patients; however, it holds limited utility in post-transplant diagnosis [2]. Quantitative DNA PCR or pp65 antigenemia assays, with PCR being the preferred modality due to its higher sensitivity and ability to quantify the viral load, are employed for routine CMV screening in kidney transplant recipients [7,8,9,10].

### 2.4. Diagnosis and Clinical Manifestation

Post-transplant cytomegalovirus (CMV) infection may be asymptomatic (viraemia without disease), manifest as CMV syndrome (fever, malaise, cytopenias), or cause tissue-invasive disease (nephritis, hepatitis, gastrointestinal disease, pneumonitis). The virus can also indirectly increase the risk of graft rejection, opportunistic infections, and chronic allograft dysfunction. The highest incidence of infection is observed in D+/R− transplant patients and those receiving lymphocyte-depleting induction agents such as anti-thymocyte globulin (ATG) [1,2]. There is no definitive consensus on the threshold of CMV DNAemia for pre-emptive treatment due to variability in testing platforms and specimen type. Although the majority of experts agree that higher levels of CMV DNAemia correlate with an increased risk of CMV infection, levels of 2–3.2 log_10_ IU/mL in plasma for D+/R− patients are considered appropriate to initiate treatment [11].

### 2.5. Management

Primary prevention involves the use of antiviral prophylaxis, such as valganciclovir or ganciclovir. The duration of antiviral prophylaxis with valganciclovir typically spans approximately 3 to 6 months, reducing the incidence of CMV infection and associated complications (Table 1) [11]. In cases of established CMV disease, treatment with valganciclovir or intravenous ganciclovir is generally initiated for a period of two weeks or until viral replication ceases. In severe or refractory cases, it may be necessary to reduce immunosuppression while carefully balancing the risk of rejection. Alternative antiviral agents, such as foscarnet, may be considered in instances of ganciclovir resistance, although such resistance is relatively uncommon [1,2].

## 3. Epstein–Barr Virus

### 3.1. Introduction

Epstein–Barr virus (EBV) is recognised for causing infectious mononucleosis; however, it holds significant importance in kidney transplant recipients due to its association with post-transplant lymphoproliferative disorder (PTLD), a potentially life-threatening complication of transplantation. EBV is widespread, with a seroprevalence reaching 90% among adults. Nonetheless, primary EBV infection can occasionally be acquired from seropositive donors, especially in paediatric recipients. Understanding and managing EBV in kidney transplant patients is essential to ensure favourable transplant outcomes by preventing PTLD [12].

The discovery of Epstein–Barr virus (EBV) dates back to 1958, when pathologist Michael Anthony Epstein proposed a viral aetiology for Burkitt’s lymphoma based on the tumour’s geographic clustering pattern [13]. The significant advancement occurred when Epstein, together with Yvonne Barr and Bert Achong, successfully identified the virus particles in cultured Burkitt’s lymphoma cells using electron microscopy [13,14]. Epstein–Barr virus (EBV) is prevalent worldwide, with infection patterns varying according to geographic and socioeconomic factors. Serological evidence indicates that up to 95% of adults in developed nations have current or past EBV infection [12]. The overall incidence of post-transplant lymphoproliferative disorder (PTLD) in adult kidney transplant recipients ranges from 1% to 2%; however, this statistic does not account for the disparities observed in specific donor–recipient serostatus combinations. The rate of PTLD reaches 22% within three years post-transplantation among the highest-risk group—EBV-negative recipients (R−) receiving kidneys from seropositive donors (D+). This situation signifies more than a sixfold increase in risk compared to other serostatus combinations [15].

### 3.2. Pathogenesis and Screening

Epstein–Barr virus (EBV) is classified within the order Herpesvirales, family Orthoherpesviridae, subfamily Gammaherpesvirinae, and genus Lymphocryptovirus [12,16]. It is also referred to as human herpesvirus 4 (HHV-4). The virus contains a linear, double-stranded DNA genome. It primarily infects B lymphocytes and epithelial cells via glycoprotein complexes, specifically gHgL-gp42 for B cells and gHgL for epithelial cells, thereby facilitating infection across multiple cell types. EBV undergoes a latent phase characterised by limited gene expression within memory B cells, enabling persistent infection, as well as a lytic phase involving comprehensive gene expression that results in disease and viral shedding [16,17]. Immunosuppression impairs cytotoxic T cell surveillance in kidney transplant recipients, thereby enabling uncontrolled proliferation of B cells. This process can result in mutations in oncogenes and tumour suppressor genes, ultimately leading to the development of Post-Transplant Lymphoproliferative Disorder (PTLD), either through primary infection or reactivation of latent infection [12].

### 3.3. Risk Stratification and Screening

Antiviral capsid antigen (VCA) IgG and IgM, along with Epstein–Barr nuclear antigen (EBNA) IgG antibodies, form the basis for determining Epstein–Barr virus (EBV) serostatus and pre-transplant risk stratification in screening protocols. VCA IgG and EBNA IgG indicate prior infection and immunity, whereas VCA IgM may suggest recent infection; however, it can produce false positives, thus necessitating clinical correlation and often EBV DNA testing [18]. Post-transplant surveillance involves quantification of EBV viral load in peripheral blood mononuclear cells or plasma via PCR. Elevated viral load predicts Post-Transplant Lymphoproliferative Disorder (PTLD), although its positive predictive value is limited [12].

### 3.4. Diagnosis and Clinical Manifestation

Asymptomatic Epstein–Barr virus (EBV) viraemia is prevalent among transplant recipients. It presents a significant challenge in distinguishing low-level viraemia from early infection that could potentially progress to Post-Transplant Lymphoproliferative Disorder (PTLD) [12,19]. Approximately 2.6% of kidney transplant recipients exhibit chronic high viral loads, with 70% of this cohort eventually clearing EBV without advancing to PTLD [19]. The clinical manifestations often mimic other post-transplant complications, such as Cytomegalovirus (CMV) infection, graft rejection, drug toxicity, or bacterial infections; therefore, maintaining a high index of suspicion is imperative [12]. The World Health Organisation (WHO) classifies PTLD into early lesions, polymorphic, monomorphic, and Hodgkin lymphoma [20]. Diagnosis is confirmed through in situ hybridisation or immunohistochemistry on biopsy specimens. Guidelines recommend re-screening EBV-seronegative candidates every 6–12 months during the transplant waiting period to prevent misclassification [18].

### 3.5. Management

Initial PTLD treatment involves reducing immunosuppression, which may induce remission in early lesions and polymorphic PTLD; however, it also increases the risk of rejection [21,22]. Rituximab monotherapy is considered the standard therapy for CD20-positive PTLD following immunosuppression, with a response rate ranging from 44% to 79% and a complete remission rate of 28% to 66%. Prolonged treatment enhances the likelihood of remission in partial responders. There exists a significant relapse rate, approximately 26% to 50%, particularly among high-risk patients who might benefit from earlier chemotherapy intervention. In cases that are resistant or exhibit aggressive behaviour, the combination of rituximab and chemotherapy (R-CHOP) remains the standard approach [21,22,23]. Adoptive T cell therapy utilising EBV-specific T cells demonstrates potential for refractory cases but is limited by availability. Additional innovative strategies include the use of monoclonal antibodies, inhibitors targeting B-cell signalling pathways, and immune checkpoint therapies such as pembrolizumab and ibrutinib [24]. Long-term surveillance through clinical assessments, laboratory tests, and imaging modalities is essential for detecting recurrence and managing associated complications [23,24].

## 4. BK Polyomavirus

### 4.1. Introduction

BK polyomavirus (BKV) affects 5–10% of kidney transplant patients. It leads to graft failure in up to 50% of untreated cases, representing a significant cause of allograft dysfunction and loss in this cohort [25,26]. The virus exhibits a recognisable progression pattern, advancing from viruria to viraemia and ultimately leading to biopsy-confirmed nephropathy [27,28]. Early identification through systemic screening and the timely reduction of immunosuppressive therapy, while carefully balancing the risk of rejection, remains essential for the management of BKV infection in kidney transplant recipients [25,29]. BK polyomavirus was first identified in urine samples obtained from a kidney transplant recipient with the initials “B.K.” in 1971 by Gardner and colleagues [25,30]. The discovery occurred concurrently with JC polyomavirus, marking the identification of the first two naturally human-tropic polyomaviruses in immunocompromised patients [30,31]. In 1993, BKV was histologically confirmed to cause nephropathy, with a notable increase in incidence observed under tacrolimus and mycophenolate-based immunosuppressive regimens [25,32]. BK virus (BKV) has a global distribution, with primary infection typically occurring during childhood via mucosal contact through oral, gastrointestinal, or respiratory routes [25,33]. Infection rates range from 60 to 100% by age 4 years, with up to 90% of adults harbouring latent virus in renal cells [25,28]. Viruria is observed in 30 to 50% of kidney transplant recipients within the first year post-transplant [27,28,33]. Approximately half of these cases progress to viraemia within 2 to 6 weeks, and 50% of viraemic recipients develop nephropathy, resulting in an incidence rate of 5 to 10% of BKV-associated nephropathy (BKVAN) [25,33]. The peak incidence generally occurs within the first six months following transplantation, with 49.3% diagnosed during this period. Nonetheless, continued vigilance is imperative to monitor for late-onset disease, which often manifests several years after transplantation [27,28].

### 4.2. Pathogenesis and Screening

BKV remains dormant in renal tubular and urothelial cells following primary infection, as cellular immunity suppresses the virus [25]. However, immunosuppressive therapy in kidney transplant recipients disrupts this equilibrium, leading to viral reactivation and proliferation [25,27]. The infection process initiates with reactivation in tubular epithelial cells, resulting in viral shedding in urine (viruria). Continued viral replication causes tumour cell lysis and inflammation, facilitating the entry of viral DNA into the peritubular capillaries and systemic circulation (viraemia), which ultimately results in nephropathy through extensive virus-induced tubular damage and interstitial inflammation [25,34].

### 4.3. Risk Stratification and Screening

Current guidelines recommend employing quantitative PCR techniques for BK virus (BKV) screening in all kidney transplant recipients [25,35]. Monthly urine and plasma BKV DNA testing are performed during the first year post-transplant, with continued screening every three months during the second year [28,36]. Urine screening demonstrates superior sensitivity for detecting early viral reactivation, whereas plasma testing provides a better correlation with clinically significant nephropathy [28,35]. The integration of urine and plasma screening protocols enhances sensitivity and specificity in identifying BKV infection among kidney transplant recipients [28,37]. The screening thresholds differ by institution, yet urine viral loads exceeding 7 log_10_ copies/mL are predictive of progression to viraemia, while plasma levels surpassing 4 log_10_ copies/mL indicate a high risk of nephropathy [25,37]. Low-level viraemia can be identified through sophisticated molecular assays such as droplet digital PCR [37,38].

### 4.4. Diagnosis and Clinical Manifestation

BKV infection is generally asymptomatic; however, increased creatinine levels and allograft dysfunction are the primary indicators of BKV-associated nephropathy [25]. Systemic symptoms caused by BKV are infrequent, thereby underscoring the importance of laboratory screening for accurate diagnosis [25,33]. The detection of “decoy cells” in urinary cytology may indicate active infection but does not possess sufficient specificity for nephropathy [25,35]. A conclusive diagnosis requires histopathological confirmation via biopsy, demonstrating cytopathic changes in tubular epithelial cells, viral inclusion bodies, and positive SV40 immunohistochemistry [25,38].

### 4.5. Management

The primary approach in management involves a meticulous reduction of immunosuppression, particularly by de-escalating or discontinuing antimetabolite agents and decreasing the dosage of calcineurin inhibitors, with viral clearance observed in 60–80% of cases. However, this strategy entails a risk of allograft rejection, necessitating a careful and balanced approach [25]. Adjunctive therapies such as leflunomide, cidofovir, fluoroquinolones, and intravenous immunoglobulin may be employed in patients who do not respond adequately to immunosuppression reduction alone; nevertheless, there exists variability in their efficacy and potential nephrotoxicity in some instances [25,36,39]. Emerging therapeutic modalities, including virus-specific T cells and monoclonal antibodies, are currently under investigation. Vigilant monitoring for rejection or recurrence remains essential in managing BK virus infection in kidney transplant recipients [36,39].

## 5. Varicella Zoster Virus

### 5.1. Introduction

Varicella zoster virus (VZV) poses a considerable risk of morbidity and mortality among kidney transplant recipients, with an incidence rate approximately 4.5 times higher than that observed in the general population [40,41,42]. Beyond localised dermatomal manifestation, there is an elevated risk of visceral dissemination, neurological complications, and systemic infections that may be life-threatening, with mortality rates reaching up to 30% in cases of disseminated disease within this patient group [43,44,45].

The understanding of the varicella zoster virus has progressed over the course of more than a century through systematic observation, research, and groundbreaking discoveries. During the 19th century, varicella (commonly known as chickenpox) was distinguished from smallpox for the first time since ancient times [46]. In 1875, Rudolf Steiner demonstrated the infectious nature of varicella by inoculating volunteers with vesicular fluid from infected individuals. In 1888, the association between varicella and herpes zoster was initially proposed, suggesting that both infections were caused by the same pathogen at different stages of manifestation. This hypothesis was subsequently validated by Thomas Weller in 1954, when he successfully isolated VZV from vesicular fluid utilising cell culture techniques [46,47]. Over 95% of adults demonstrate evidence of prior VZV infection, indicating a nearly universal epidemiology of the virus [40,42,48]. Among kidney transplant recipients, VZV incidence peaks within the first year, with a median of five months, yet remains elevated, with cases reported up to thirteen years post-transplant. Patients aged over sixty face a fourfold increase in VZV risk [48]. Approximately three to five per cent of kidney transplant recipients are VZV-seronegative and are consequently at the highest risk of potentially life-threatening, severe primary infection [42].

### 5.2. Pathogenesis and Screening

The pathogenesis of VZV in kidney transplantation involves intricate interactions between viral latency, immune surveillance, and immunosuppression [49,50]. Following primary infection, VZV remains latent in sensory ganglia such as the trigeminal, autonomic, and dorsal root ganglia, with the viral genome persisting as episomal DNA in neurons, exhibiting minimal gene expression [50]. Continuous immune surveillance by CD8+ cytotoxic T cells sustains latency. Factors such as ageing, stress, trauma, or immunosuppression influence immune control, facilitating reactivation via the nerves to the skin, resulting in a vesicular rash [49,50]. Immunosuppressive agents such as mycophenolate mofetil, anti-thymocyte globulins, and calcineurin inhibitors have been associated with an increased risk of VZV reactivation [43,44,45,51]. The severity of infection correlates with the level of immunosuppression, with 8.6% of fulminant cases occurring in kidney transplant recipients under intense immunosuppression [51].

### 5.3. Risk Stratification and Screening

Universal serology testing identifies VZV-seronegative patients, in whom vaccination is crucial [52]. Enzyme-linked immunosorbent assays detect IgG antibodies to VZV, the presence of which indicates prior infection or immunity, though immune responses may decline in immunocompromised hosts [53]. False negatives do occur in the context of immunosuppression or recent immunoglobulin treatment [52,53]. The live varicella vaccine should be administered at least four weeks before transplantation, but it is contraindicated post-transplant due to the risk of infection [52]. The recombinant zoster vaccine presents a safe alternative for immunocompromised patients [53]. Routine VZV viral load assessment is not standard in asymptomatic patients and is primarily reserved for monitoring infection in high-risk patients during herpes zoster prodrome [54]. Monitoring VZV DNA is essential following exposure or in cases presenting neurological symptoms, prolonged pain, or atypical rashes, to evaluate immunity and inform prophylactic strategies [43,54].

### 5.4. Diagnosis and Clinical Manifestation

Varicella zoster virus (VZV) infection in kidney transplant recipients is more severe and broader compared to immunocompetent hosts [42,43,44]. Classical dermatomal herpes zoster occurs in 90% of cases, while atypical presentations may involve non-dermatomal distribution, absence of pain, or lack of rash [43]. Disseminated zoster affects approximately 10 to 15.9% of patients, presenting with widespread lesions and associated high morbidity, with a mortality rate reaching up to 30% [42,43,44]. It may involve any organ system, occasionally without cutaneous manifestations, leading to conditions such as pneumonia, hepatitis, or encephalitis [45,55]. Secondary bacterial infections are notable, potentially resulting in multiorgan failure and death in severe disseminated cases [42,45]. Modern diagnostic approaches for VZV rely primarily on molecular techniques, notably polymerase chain reaction (PCR) testing of lesional specimens. PCR analysis of vesicle fluid or swabs allows for rapid and precise diagnosis [54,55]. Real-time PCR detection of VZV DNA correlates with disease severity. Plasma or serum PCR testing is valuable in cases of visceral disease lacking skin lesions [45,54]. Next-generation sequencing proves helpful in complex clinical scenarios, while serological testing, although limited for acute diagnosis, is beneficial for confirming immunity or seroconversion status [45,55].

### 5.5. Management

Vaccination remains the most effective method for preventing VZV infection in kidney transplant recipients, emphasising the importance of pre- and post-transplant immunisation [52,53]. Varicella zoster immune globulin (VariZIG) is administered concurrently with oral acyclovir within 96 h post-exposure in seronegative recipients, whereas significant exposure in seropositive recipients may necessitate prophylactic acyclovir [56,57]. Oral acyclovir (800 mg five times daily) is employed for the treatment of localised disease, while intravenous acyclovir (10–15 mg/kg) is reserved for severe cases. It is imperative to commence treatment within 72 h of rash onset [42,45,56]. Valacyclovir (1000 mg three times daily for seven days) presents a suitable alternative, with dose adjustments required for all cases involving renal impairment [56,58]. Disseminated VZV necessitates prompt hospitalisation, intravenous therapy, and supportive care, including intensive care unit management, alongside reduction of immunosuppression [42,43]. Approximately 16% of kidney transplant recipients experience recurrence of VZV infection; therefore, long-term prophylaxis with low-dose acyclovir or valacyclovir may aid in preventing recurrence [58].

## 6. Adenovirus

### 6.1. Introduction

Adenovirus typically causes mild infections, most commonly affecting the lower respiratory tract, gastrointestinal tract, or conjunctiva. Adenovirus is a common cause of upper respiratory tract infections, resulting in 5–10% of paediatric and 1–7% of adult respiratory tract infections [59]. It is a rare cause of mortality; however, in patients with reduced cellular immunity, it can result in severe presentations, including pneumonia and graft dysfunction. It remains a significant cause of morbidity and mortality in the renal transplant cohort.

### 6.2. Pathogenesis and Screening

Adenoviruses are non-enveloped icosahedral viruses with a double-stranded DNA genome [60]. About 67 serotypes are known to be pathogenic to humans. Different adenovirus subtypes can cause various disease states affecting the respiratory tract, gastrointestinal tract, urinary tract, or eyes. Infection is usually transmitted through respiratory droplets or ocular secretions [61]. Adenovirus replicates in host cells, leading to a lytic infection that causes cytolysis and inflammation. It can also cause latent infections where the virus resides in lymphoid tissue. The host immune response produces pro-inflammatory cytokines that can cause cellular damage and even a cytokine storm, contributing to severe disease presentations such as pneumonitis [61,62].

Detection of adenovirus is by culture or PCR testing. PCR testing has become widely available and largely replaced viral culture and older diagnostic methods [63]. PCR is a rapid and highly sensitive method that can detect adenovirus at 100 to 1000 copies/mL. Blood, respiratory, tissue, and stool specimens can be used for PCR testing. Serial quantitative PCR can be used to guide therapy initiation. Persistently elevated or rising viral loads (0.5–1.0 log increase) may signal a need for intervention [64,65]. Results from molecular assays should be correlated with histopathology, where available, and clinical presentation to distinguish between asymptomatic infection and disease. Histopathologic diagnosis remains the gold standard for invasive disease [66].

Under light microscopy, smudgy basophilic intranuclear inclusions with enlarged nuclei are seen in infected cells [67]. Distal tubules are usually more affected than proximal tubules. There is often associated acute tubular injury, frequently with tubular necrosis. Interstitial nephritis, often with a pleomorphic infiltrate, features interstitial oedema and haemorrhage. The destruction of tubules may be linked to necrotising interstitial granulomas. Severe granulomatous tubulointerstitial nephritis is considered characteristic of adenoviral infection. Immunostaining for adenovirus shows strong nuclear and cytoplasmic staining in infected cells [67]. Specific staining is observed on immunofluorescence. Electron microscopy reveals viral particles in the nuclei of infected cells. 

Surveillance is not currently recommended in asymptomatic individuals with a solid organ transplant [66].

### 6.3. Diagnosis and Clinical Manifestation

Adenovirus infections can range from asymptomatic to a severe, prolonged disease course. Clinical manifestations depend on the site of infection and the type of transplanted organ. Kidney transplant patients can have haematuria, dysuria, fever, haemorrhagic cystitis, and graft dysfunction [64,68,69]. Common extra-renal manifestations include orchitis, gastroenteritis, and pneumonia [64,70]. Significant risk factors for adenovirus infection include younger age (<5 years old) [71,72], degree of immunosuppression [64,66,71] and type of organ transplant, with intestinal transplant conferring the most considerable risk [66,71].

### 6.4. Management

The mainstay of therapy for adenovirus is supportive care and a reduction in immunosuppression [73]. The role of immune recovery is highlighted by the resolution of viraemia following the reduction in immunosuppression [64,74]. At present, there is no specific antiviral drug approved for use in the treatment of adenovirus infection due to a lack of supportive clinical trial evidence [17]. Cidofovir and ribavirin use have been supported by case reports [75,76,77,78]. Cidofovir is a nucleotide analogue of cytosine that inhibits viral DNA polymerase. It is associated with nephrotoxicity and neutropenia [66,79]. Two commonly used regimens include 1 mg/kg/week or 5 mg/kg/week for 2 weeks, followed by 5 mg/kg every other week until resolution [66]. The dose should be adjusted based on renal function, with adults with creatinine clearance < 50 mL/min, reducing the dose to 0.5 mg/kg/week. Probenecid should be administered 3 h before, 2–3 h and 8 h after as it competes with probenecid at the proximal tubule to reduce nephrotoxicity [66]. To overcome some of the issues with cidofovir, brincidofovir was developed as a lipid ester prodrug of cidofovir. It is currently approved for the treatment of smallpox and is undergoing clinical trials in adenovirus [80].

Ribavirin does not appear to have significant anti-adenovirus activity in humans and is not recommended for use to treat serious adenoviral infections [73].

## 7. Parvovirus

### 7.1. Introduction

Parvovirus B19 is best known as the causative pathogen in fifth disease, which is a self-limiting illness that affects children. It is a small non-enveloped single-stranded DNA virus of the family Parvoviridae, the subfamily Parvovirinae, and the genus Erythrovirus [81]. It was discovered in 1975 from sera obtained from studies on hepatitis B, but it was not until 1981 that the infection was associated with aplastic crisis [82]. Its natural course is varied amongst renal transplant recipients, but its most common manifestation is anaemia. It has also been implicated in pancytopaenia, hepatitis, myocarditis, neurological disease, and even allograft dysfunction [83,84,85,86,87]. Parvovirus B19 infection tends to peak in childhood, with seroprevalence peaking in adulthood [88].

### 7.2. Pathogenesis and Screening

Acquisition of parvovirus occurs in the respiratory tract; it is presumed to replicate in nasopharyngeal tissue [89]. Parvovirus B19 targets human erythroid progenitor cells in the bone marrow, particularly burst-forming unit-erythroid cells and colony-forming unit-erythroid cells. Erythroid progenitor cells produce infectious virus and are destroyed, leading to a drop in circulating reticulocytes, which can last 2–5 days [89]. There are three distinct genotypes of Parvovirus B19; genotype 1 is the predominant circulating genotype. Genotype 2 is largely ancestral to genotype 1 and is detected in older patients. Genotype 3 is limited to sub-Saharan Africa and South America. No correlation between disease symptoms and genotype has been detected [90].

### 7.3. Diagnosis and Clinical Manifestation

Diagnosis of parvovirus B19 can be conducted via molecular measurements or viral antibodies. Viral DNA can be detected in the blood, bone marrow or infected organs using polymerase chain reaction (PCR) [56]. PCR remains the gold standard for diagnosis and has significantly improved detection rates for Parvovirus B19 [91]. However, PCR may not detect non-B19 strains (genotypes 2 and 3), and real-time PCR can be negative in the setting of high viraemia [92]. PCR may remain persistently positive for an extended period of time, long after the acute infection has resolved; however, its positive predictive value remains high in the setting of aplastic anaemia [93]. IgM assays can detect a recent infection, but their reliability is reduced due to a delayed humoral response, which may be exacerbated in immunosuppressed individuals [94]. Parvovirus B19 was only present in 75% of solid organ recipients at the time of diagnosis [83]. If serology and PCR are negative but suspicion remains high, then a bone marrow biopsy with immunohistochemical staining can help establish the diagnosis [87]. The main histopathological features on kidney biopsy are thrombotic microangiopathy and collapsing glomerulopathy [95,96,97]. At present, there is no guidance recommending screening for parvovirus B19 in kidney transplant recipients.

### 7.4. Management

The mainstay of treatment for parvovirus B19 is reduction of immunosuppression and IVIg [98]. However, efficacy data for this strategy have come mainly from case reports. There have not been any clinical trials performed demonstrating efficacy. The optimal dose of IVIg has not been established, but current guidance recommends that patients be treated with 400 mg/kg/day [99]. Relapse remains common post-treatment, with reports as high as 28% [87]. In the same case series, the rate of relapse was unchanged among transplant recipients who received a dose of ≤2 g/kg or >2 g/kg. Immunosuppression should be reduced at the time of diagnosis [99]. The role of PCR in monitoring response to treatment is unclear; current guidance recommends monitoring serial haemoglobin measurements and repeating a PCR in the event of recurrence of anaemia [99]. In the event of non-response to an initial course of IVIg or relapse, then a further course of 400 mg/kg/day of IVIg can be considered.

## 8. Sapovirus

### 8.1. Introduction

Sapovirus is named after an outbreak of diarrhoeal illness in children in Sapporo, Japan, in 1977 [100]. Sapoviruses are single-stranded RNA viruses first identified in humans in 1976 by electron microscopy [101]. Sapovirus typically results in an acute diarrhoeal illness that is self-limiting for the majority of immunocompetent adults. It is a significant cause of infant diarrhoea, accounting for 24.7% of diarrhoeal cases [102]. Overall, sapovirus prevalence is estimated at 3.4%, with the highest prevalence amongst children < 5 years of age [103].

### 8.2. Pathogenesis and Screening

Sapovirus is transmitted through the faecal-oral route with a typical incubation period of one to four days. Sapovirus infection can have prolonged duration and increased severity of symptoms in transplant recipients due to impaired mucosal immunity and dysfunction of secretory IgA responses. Sapovirus is diagnosed by stool sampling using Multiplex real-time PCR [104].

### 8.3. Diagnosis and Clinical Manifestation

All kidney transplant recipients with sapovirus infection present with watery diarrhoea, nausea, vomiting and abdominal cramping. Sapovirus is an important cause of chronic gastroenteritis in immunocompromised individuals [105]. Acute gastroenteritis is a leading cause of presentation for patients with a renal transplant to the emergency department [106]. A significant deterioration in renal function constitutes the most critical complication associated with sapovirus infection in kidney transplant recipients. Additionally, elevated serum tacrolimus concentrations were observed in kidney transplant recipients infected with sapovirus, potentially indicating altered tacrolimus pharmacokinetics during episodes of acute diarrhoeal illness [107].

### 8.4. Management

General prevention remains the cornerstone in managing sapovirus, given that no vaccine is presently available. Maintaining good hygiene, especially through regular handwashing and measures to prevent faecal-oral transmission, is essential for effective prevention [107]. No specific antiviral treatment is recommended for sapovirus, and current management is supportive. Nitazoxanide is an emerging treatment for sapovirus infection in the renal transplant cohort; however, its use is currently only supported by case reports [107,108,109].

## 9. Mpox Virus

### 9.1. Introduction

Mpox, previously known as monkeypox, is an infection that was first described in humans in the 1970s [110]. The first case was described in the Democratic Republic of the Congo and was previously thought to be a disease confined to the African continent [111]. However, in 2024 the World Health Organisation (WHO) declared a monkeypox outbreak as a public health emergency internationally with over 100,000 laboratory-confirmed cases between June 2022 and August 2024 across more than 120 countries with over 220 deaths [112]. Unsurprisingly, there has been concern about how this outbreak may impact renal transplant recipients with multiple case reports and case series examining the impact of Mpox in the transplanted population [113,114,115,116,117,118,119].

### 9.2. Pathogenesis and Screening

Mpox is an orthopoxvirus in the Poxviridae family with multiple modes of transmission: via infected animals through exposure to bodily fluids or consumption of undercooked meat or animal bites, via human-to-human transmission through direct contact with respiratory droplets or sexual contact and via vertical transmission from infected mothers to newborns [120]. Theoretically, Mpox could be transmitted via organ donation from an infected donor. While no cases have been reported to date from transplantation, in anticipation of the clinical concern, the Organ Procurement and Transplantation Network (OPTN) released guidance on the screening of high-risk donors [121]. Post-transplantation viral screening is not protocolised for Mpox and is performed based on clinical suspicion.

### 9.3. Diagnosis and Clinical Manifestation

Mpox is diagnosed primarily by real-time polymerase chain reaction (real-time PCR) performed on material taken from skin lesions [112]. Mpox should be considered in those presenting with fever, myalgias, sore throat, headache and lymphadenopathy. The classical skin lesions are described as papules that can progress to vesiculopustular eruptions and the mucosal lesions can be seen as chancriform ulcers, oropharyngeal ulcers and proctitis [113]. Severe manifestations of the virus can result in ocular disease, encephalitis, pneumonitis and secondary bacterial infections [122]. There appears to be an increased mortality risk for those immunosuppressed, particularly with HIV, those who contract the virus in infancy and those infected with Clade 1 Mpox virus [122]. Published case series and case reports on Mpox in solid organ transplant recipients vary significantly from mild, self-limiting manifestations to severe disease resulting in intensive care admissions and even a reported fatality [113,117,118]. Transplant recipients with HIV are a particularly high-risk cohort for Mpox infection [113,118].

### 9.4. Management

Mpox treatment strategies have been borrowed from smallpox [123]. The CDC has recommended the use of antiviral agents in high-risk patients, including kidney transplant patients. Therapy options are limited by a lack of substantial evidence in human studies or focused Mpox studies, instead relying on translation from smallpox studies and animal studies. Current strategies used alongside best supportive therapy include [124]:

- Tecovirimat blocks the viral protein VP37 and inhibits the production of extracellular virus. It is the current recommended first-line therapy for the treatment of Mpox and has been used successfully in select renal transplant patients but requires close monitoring of Tacrolimus levels (as it is an inducer of the CYP3A4 cytochrome) and renal function as it has been associated with acute kidney injury [115,116,123,125].
- Cidofovir is a drug that once phosphorylated inside the cell acts to inhibit viral DNA polymerase [112]. It is unfavourable in renal transplant patients due to the association with nephrotoxicity and with the Fanconi syndrome [126].
- Brincidofovir is a prodrug of cidofovir less associated with nephrotoxicity but associated with hepatotoxicity [123]. It has been used to successfully treat Mpox in transplant recipients without significant reported adverse events [127].

Vaccination:

- The CDC has clear guidance on who is eligible for vaccination based on risk factor profile [128]. There are two vaccines available: a non-replicating smallpox and mpox vaccine (JYNNEOS, IMVAMUNE, IMVANEX) or a live attenuated vaccine (ACAM2000). The non-replicating vaccine can be safely used in transplant patients [123,129].
- Vaccinia immunoglobulin is available as post-exposure prophylaxis and is safe in transplant patients [123,129].

## 10. Pegivirus

### 10.1. Introduction

Human Pegivirus 1 (HPgV1) was first discovered in 1995 and was historically known as Hepatitis G virus or GB virus Type C. HPgV-1 is highly prevalent globally with estimates that one-sixth of the global population are seropositive for the virus [130].

### 10.2. Pathogenesis and Screening

HPgV-1 is a single-stranded RNA virus of the Pegivirus genus, which is a member of the Flaviviridae family [130,131,132]. HPgV-1 is considered a blood-borne virus and is transmitted by parenteral or sexual contact and vertically from mother to infant [130,132,133]. Haemodialysis patients have a higher risk of infection via parenteral exposure, and this risk is carried through post kidney transplantation [131]. There is no established routine screening for the virus in transplantation.

### 10.3. Diagnosis and Clinical Manifestation

HPgV-1 is diagnosed by serological testing for viral RNA by polymerase chain reaction [134]. Viral clearance can be demonstrated by antibodies to the major viral envelope glycoprotein [135]. Testing for HPgV-1 is not widely or commercially available. To date there has been no definitive evidence that HPgV-1 has a pathogenic role in humans and in small studies in the transplant population there appeared to be no impact on patient outcomes [131,132,133]. However, HPgV-1 and its impact on the immunosuppressed transplant population are an area of expanding research with some studies and case reports showing an association with lymphoma and with leukoencephalitis and myelitis presenting with progressive neuropathy and progressive myelopathy [130,136]. In contrast, it has also been studied as a potentially beneficial co-infection for those with HIV and Hep C with reported improved outcomes likely owing to the immunomodulatory role of the HPgV-1 virus particularly on T lymphocytes [130]. This benefit has not been shown in the transplant population [133].

### 10.4. Management

There is no established treatment for HPgV-1 in the transplant population. Case reports that have demonstrated HPgV-1 in the CSF of patients with ‘Pegivirus-associated encephalomyelitis’ have trialled different therapeutic approaches such as interferon alpha, ribavirin or reducing/stopping immunosuppression, but these were not evidence-based or established interventions; these were efforts to treat people whilst critically unwell [136].

## 11. Human Papillomavirus

### 11.1. Introduction

Human papilloma virus (HPV) was initially discovered in the early 1900s and was first linked to skin warts. It wasn’t until the 1980s and the work of Harold zur Hausen that the association between HPV 16 and 18 and cervical cancer was established, and his discovery earned him the Nobel Prize in Physiology and Medicine in 2008 [137]. HPV infection poses a significant risk to the general population, as the most common sexually transmitted infection worldwide with estimates that the lifetime risk of acquiring HPV ranges from 50–100% [138]. The risk of acquiring HPV infection is increased in solid organ transplant recipients [139].

### 11.2. Pathogenesis and Screening

HPV is a small, non-enveloped DNA virus and member of the Papillomaviridae family [140]. HPV viruses can be divided into low-risk or high-risk subtypes based on the associated cancer risk with HPV 16 and 18 being classically high-risk and HPV 6 and 11 being low risk [141]. HPV can be transmitted sexually, vertically from mother to infant and through direct or indirect contact with infected individuals [140]. Approximately 139 countries worldwide have implemented official cervical cancer screening programmes for the general population and 48 of these countries use HPV testing as part of the cervical screening process [142]. In the transplant population it is recommended that screening intervals are shortened in line with individuals with HIV infection [139]. It is recommended that in the first year post kidney transplant, cervical screening is performed 6-monthly, and then moved to annual surveillance. HPV testing is incorporated into the post-transplant cervical screening algorithm with some centres increasing the frequency of screening in those testing positive for high-risk HPV [139,141].

Screening for HPV virus is most commonly performed in cervical cancer but in high-risk populations, i.e., those engaging in receptive anal intercourse, anal cancer screening has been recommended in the transplant population [139].

### 11.3. Diagnosis and Clinical Manifestation

HPV can present in a variety of ways in the kidney transplant population; cutaneous warts, anogenital warts, respiratory papillomatosis, premalignant lesions of the cervix and anus, malignant lesions of the cervix, anus, vulva, penis, and oropharynx [139,141]. Diagnosis of HPV related disease is made by local examination of the respective area with samples taken for cytological assessment and molecular detection of HPV. HPV DNA testing has been recommended by the WHO as preferable to cytology or clinical examination of the cervix as DNA testing is proven to be more sensitive in detecting cervical precancers and cancers, but this recommendation is yet to be adopted into most international practices [142].

### 11.4. Management

The treatment of HPV related disease in the transplant population focuses on three main areas; 1. HPV prevention, 2. Local management determined by the type of lesion, the grade and location and 3. Immunosuppression review [141]. Vaccination options include a bivalent, quadrivalent or nonavalent vaccine. Ideally vaccination should be given early, prior to sexual activity, and pre-transplant but it can be given safely post-transplant [139,143]. Local therapy strategies are lesion dependent. Cutaneous warts can be treated with topical therapies such as salicylic acid and imiquimod cream or with cryotherapy. If there is insufficient response to these therapies, they warrant specialist dermatology input. Pre-malignant cervical lesions can be treated by local excision or ablation. Treatment of cervical cancer is stage dependent with surgical and chemo-radiation therapy guided by gynae-oncology. Premalignant anal lesions can be treated with topical therapies such as trichloroacetic acid, 5-fluorouracil, imiquimod and cryotherapy [139]. Treatment of anal lesions requires careful consideration of grade and risk assessment as more invasive treatments for larger, higher-grade lesions can lead to complications such as pain, incontinence and stenosis [139]. Malignant anal lesions are usually treated with combined-modality therapy, combination radiation therapy, and chemotherapy, as surgical options often require creation of a permanent colostomy post-abdominoperineal resection and removal of the anorectum [139]. In cases of refractory or recurrent disease, immunosuppression may be reduced or altered with some studies showing a benefit of class switching from calcineurin inhibitors to mTOR inhibitors [144,145].

## 12. Hepatitis B

### 12.1. Introduction

Hepatitis B (HBV) infection continues to be a major cause of chronic liver disease and hepatocellular carcinoma worldwide and is particularly prevalent in the haemodialysis population due to the significant exposures encountered during treatment such as constant needling, exposure to shared dialysis machines and blood products [146,147]. HBV infection in transplantation is a multifaceted topic requiring consideration of preventative strategies in patients with chronic kidney disease, treatment and prevention strategies in recipients who have had HBV who will be immunosuppressed and the risk of reactivation and now more recently HBV infected donors owing to the implementation of “extended donor criteria organs” in an effort to reduce transplant wait lists particularly in endemic areas [148].

### 12.2. Pathogenesis and Screening

HBV belongs to the Hepadnaviridae family and is a partially double-stranded DNA virus with a spherical double-shelled structure of approximately 42 nm in diameter [146]. As it is a blood−borne virus it can be transmitted vertically from mother to infant or horizontally via exposure to contaminated blood including from donor to recipient with kidney transplantation [149].

Screening for HBV is an important part of chronic kidney disease management and the pre-transplant assessment. Potential kidney transplant recipients should undergo serological testing for Hepatitis B surface antigen (HBsAg), Hepatitis B surface antibody (anti-HBs) and hepatitis B core antibody (anti-HBc) [147]. Potential kidney donors (both living and deceased) require testing for HbsAg, anti-HBc and HBC DNA nucleic acid testing (NAT) [150].

### 12.3. Diagnosis and Clinical Manifestation in Transplant

HBV is diagnosed based on serological testing (Table 2) [150]. HBV reactivation post transplantation can vary from asymptomatic infection to fulminant hepatitis and ultimately can lead to the development of cirrhosis [151]. HBsAg positive status post transplantation and the increased risk of graft failure and mortality is currently inconclusive with several recent systematic reviews and meta-analyses showing that when appropriate antiviral therapy is used there was no real difference in outcomes [152,153]. Hepatitis B is associated with various renal manifestations including membranous nephropathy, membranoproliferative glomerulonephritis and polyarteritis nodosa and these complications can occur in the transplant population [154].

### 12.4. Management

It is recommended that all patients with chronic or prior HBV infection should be treated with antiviral therapy immediately post-transplant, particularly those who are HBsAg+ or receiving B-cell depleting therapies [147,151,155]. Those with chronic infection may have their therapy continued into the post-transplant period. First-line antiviral agents in the renal transplant population include entecavir and tenofovir alafenamide [147,155]. In those with prior infection, duration of therapy can vary from indefinite to 6–12 months post-transplant, but should be withdrawn cautiously if they have stable renal function, stable low-dose immunosuppression and no evidence of HBV [147]. In those receiving a kidney transplant from HBsAg+ donors, antiviral therapy should be initiated and is usually continued for up to one year post transplant [156].

Ideally, HBV vaccination should be an early part of the chronic kidney disease assessment and management plan with completion of vaccination prior to initiation of dialysis or need for transplantation. Whilst on dialysis, most centres routinely monitor the seroresponse to the HBV vaccine and often additional doses or shorter intervals are required for an adequate response. Post-transplant vaccination is associated with a poor seroprotective response but should be given to those who are unvaccinated, usually at 3–6 months post-transplant when they are established on low-dose maintenance immunosuppression [157].

## 13. Hepatitis C

### 13.1. Introduction

Hepatitis C virus (HCV) is one of the most common chronic viral infections, with US data reporting HCV infection in 1% of the general population and 3–14% in those living with chronic kidney disease [158,159,160]. Since the emergence of direct-acting antivirals (DAAs) in the treatment of Hepatitis C the landscape of renal transplantation in patients with Hepatitis C has shifted drastically [158].

### 13.2. Pathogenesis and Screening

Hepatitis C is a small, enveloped RNA virus that belongs to the genus Hepacivirus of the Flaviviridae family. Hepatitis C is a blood-borne virus that can be transmitted vertically from mother to infant and via horizontal transmission via nosocomial exposures such as haemodialysis, blood products and kidney transplantation [158]. Screening for HCV is performed routinely in all dialysis patients and all transplant candidates at regular intervals based on the 2022 Kidney Disease: Improving Global Outcomes clinical practice guidelines (KDIGO) [161]. Initial HCV antibody testing is recommended but if positive will require further HCV RNA testing. HCV seropositive donor kidneys are being transplanted as part of the ‘extended donor criteria organs’ and screening takes place to assess both the donor and recipient HCV RNA status at transplant [162].

### 13.3. Diagnosis and Clinical Manifestation

HCV infection in the kidney transplant population has both renal and systemic manifestations. HCV infection is associated with adverse outcomes in kidney transplant recipients with evidence of increased risk of acute rejection, chronic allograft nephropathy, new onset diabetes after transplantation, HCV associated cryoglobulinaemic vasculitis, membranoproliferative glomerulonephritis and membranous nephropathy [158]. In the context of immunosuppression use post-transplant there can be decompensation or progression of HCV infection with acute on chronic flares of hepatitis, progression to cirrhosis or the development of hepatocellular carcinoma [158]. Although transplantation carries risk in the HCV infected population it remains the best option for patients versus maintenance on haemodialysis [161,163].

### 13.4. Management

In accordance with the KDIGO guidelines it is recommended that treatment of HCV be initiated in those with CKD, those receiving dialysis or post kidney transplant [161]. Choice of therapy assesses prior treatments, drug interactions, kidney disease stage, hepatic fibrosis stage, comorbidities and transplant status. Pangenotypic and genotype-specific regimens are deemed safe and effective in kidney transplant recipients [160]. Data is limited on the use of DAAs in those with GFR < 30 mL/min/1.73 m^2^ but is still recommended with caution. Post- transplant therapy options include Glecaprevir/pibrentasvir, ledipasvir/sofosbuvir, and velpatasvir/sofosbuvir, elbasvir/grazoprevir and sofosbuvir/velpatasvir/voxilaprevir [158]. Close attention is needed when prescribing DAAs alongside transplant immunosuppression as there are a number of drug-drug interactions [160]. In those patients who receive a HCV positive donor kidney various therapeutic strategies have been tested including pre-emptive treatment once HCV RNA was detectable or prophylactic approaches with first dose DAA in the operating room with favourable outcomes. Use of HCV positive donor kidneys appears safe and well tolerated [158,164].

Post-transplant, patients require continued assessment of HCV status with 3 monthly NAT testing and liver function assessment. Those who were diagnosed as having cirrhosis pre-transplant should continue routine hepatology follow up including serial liver ultrasounds.

It is imperative that haemodialysis units adhere to strict infection control practices for the prevention of HCV transmission [160].

## 14. Discussion

Several limitations hinder the translation of viral transplant research into standard nephrology practices. Most evidence on screening intervals, viral load thresholds, and treatments comes from heterogeneous observational studies, single-centre studies, and retrospective registries rather than randomised trials. This creates variability in managing CMV, EBV, and BK virus. Differences in viral load assays, sample types, reporting, and the reduction of immunosuppression contribute to the inconsistency as well. As a result, guideline thresholds are based on consensus rather than validated outcomes across populations [11,165].

Many non-classical viral infections—such as adenovirus, parvovirus B19, sapovirus, pegivirus, and Mpox—are documented mainly through case reports and small series, often with confounding factors such as indication bias and publication bias toward severe or atypical cases. These limitations make it difficult to accurately determine incidence, graft loss, and survival impacts, thereby hindering meaningful comparisons among immunosuppression or antiviral strategies. Similar gaps are evident in the context of VZV vaccination strategies, the optimal dosing and duration of IVIG for parvovirus B19, and the emerging role of agents such as virus-specific T cells in managing BK polyomavirus and other refractory infections [52,165].

Priority areas in clinical nephrology include multicentre trials to confirm virological thresholds and develop surveillance algorithms for infections such as CMV, EBV, and BK polyomavirus, in addition to developing new therapeutics [1,165]. A summary table of viruses, including important risk factor, suggested screening technique and frequency, prophylactic and first-line treatments are available in the Supplementary Material.

## 15. Conclusions

The success of organ transplant is accompanied by an increased risk of infection, particularly viral etiologies. This chapter illustrates myriad of viruses that a kidney transplant recipient may be exposed to, in addition to a variety of manifestations that range from inconsequential to life-threatening. Transplant practitioners need to be aware of these viruses, to perform appropriate screening and detect them early to avoid progression and associated complications. Each of these viruses has nuances to its treatment; that usually requires multi-disciplinary input.

## Figures and Tables

**Table 1:** Recommended approach for CMV prevention in adult kidney transplant recipients.

Serostatus	Risk Level	Recommendation
D+/R−	High	Valganciclovir for 6 months
D−/R+	Intermediate	Valganciclovir for 3 months
D−/R−	Low	Valacyclovir for 3 months

**Table 2:** Interpretation of Hepatitis B Serology Pre-transplantation.

Serology	Interpretation	Recommendation
HBsAg −/ Anti-HBc −/ Anti HBs−	Susceptible	Vaccination
HBsAg −/ Anti-HBc +/ Anti HBs +	Prior infection (Inactive)	Monitor Anti-HBs titres +/− re-vaccinate
HBsAg −/ Anti-HBc −/ Anti HBs+	Immune due to hepatitis B vaccination	Monitor Anti−HBs titres +/− re-vaccinate
HBsAg +/ Anti-HBc +/ Anti HBs −/ IgM anti-HBc+	Acute infection	Treatment of acute infection prior to consideration of transplant
HBsAg +/ Anti-HBc +/ Anti HBs −/ IgM anti-HBc −	Chronic infection	Anti−viral therapy and proceed with transplant once no contraindications
HBsAg −/ Anti-HBc +/ Anti HBs−	Recovering (IgM antiHBc+) Recovered with yet undetectable anti-HBs Susceptible with false + anti-HBc Occult HBV	Additional testing

## Data Availability

No new data were created or analysed in this study. Data sharing is not applicable to this article.

## Supplementary Materials

The following supporting information can be downloaded at: https://www.mdpi.com/article/10.3390/jcm15031166/s1, Table S1: Summary table of viruses, including important risk factors, suggested screening technique and frequency, prophylactic and first-line treatments.
