# Supplementary material for: Viral Infections in Kidney Transplant Recipients: Current Practice and Updates
Source: J Clin Med. 2026 Feb 2;15(3):1166. doi: 10.3390/jcm15031166 (PMC12897623; doi:10.3390/jcm15031166)
Supplement: Supplementary file 1 [file jcm-15-01166-s001.zip › jcm-4055723-supplementary.pdf]

Virus	Key risk factors	Suggested screening & frequency	Prophylaxis / prevention	First-line treatment
Cytomegalovirus (CMV)	D+/R− highest risk; D−/R+ intermediate; D−/R− lowest; lymphocyte-depleting induction (ATG); high net immunosuppression.	Quantitative CMV DNA PCR (preferred) or pp65 antigenemia; monthly for first 6 months, then less frequent; pre-transplant serology for risk stratification.	Antiviral prophylaxis with valganciclovir or ganciclovir for 3–6 months (6 months D+/R−, 3 months D−/R+; valacyclovir 3 months for D−/R−).	Valganciclovir or IV ganciclovir for CMV disease, typically ≥2 weeks or until virological clearance; consider immunosuppression reduction; foscarnet for resistant cases.
Epstein–Barr virus (EBV)	EBV D+/R− (highest PTLD risk, up to 22% at 3 years); high overall immunosuppression.	Pre-transplant VCA IgG/IgM and EBNA IgG for serostatus; post-transplant EBV DNA PCR monitoring in blood; repeat serology every 6–12 months in EBV-seronegative candidates on the waiting list.	No universal antiviral prophylaxis; risk mitigation via serostatus-based stratification and careful immunosuppression.	First step is reduction of immunosuppression; rituximab monotherapy for CD20+ PTLD; rituximab plus chemotherapy (e.g. R-CHOP) for aggressive or refractory disease; adoptive EBV-specific T cells in selected refractory cases.
BK polyomavirus (BKV)	High overall immunosuppression (especially tacrolimus/mycophenolate regimens); early post-transplant period.	Quantitative PCR for BKV DNA in urine and plasma: monthly in year 1, then every 3 months in year 2; urine threshold >7 log10 copies/mL predicts viraemia, plasma >4 log10 copies/mL predicts nephropathy.	No specific antiviral prophylaxis; strategy is early detection with structured PCR surveillance and cautious immunosuppression use.	Stepwise reduction of immunosuppression (stop or reduce antimetabolite, lower CNI) clears viraemia in 60–80%; consider leflunomide, cidofovir, IVIG or fluoroquinolones in non-responders, acknowledging limited evidence and

Virus	Key risk factors	Suggested screening & frequency	Prophylaxis / prevention	First-line treatment
				nephrotoxicity risk.
Varicella–zoster virus (VZV)	Age >60; high net immunosuppression (e.g. MMF, ATG, CNIs); VZV-seronegative status; early post-transplant period.	Universal pre-transplant VZV IgG serology; VZV DNA PCR in blood or lesion samples when clinically indicated; no routine viral load surveillance in asymptomatic patients.	Pre-transplant live varicella vaccine in seronegative patients (≥4 weeks pre-transplant); post-transplant recombinant zoster vaccine (non-live); post-exposure VariZIG plus oral aciclovir in seronegative recipients; short aciclovir prophylaxis after significant exposure in seropositive patients.	Localised disease: high-dose oral aciclovir or valaciclovir with renal dose adjustment; disseminated or severe disease: IV aciclovir plus hospitalisation, supportive care and reduction of immunosuppression; consider long-term low-dose aciclovir/valaciclovir for recurrence prevention.
Adenovirus	Younger age (<5 years in general data); intense immunosuppression; certain organ types (highest with intestinal transplant, still relevant in kidney recipients).	Diagnosis by PCR on blood, respiratory, tissue or stool specimens; serial quantitative PCR to guide therapy; histopathology is gold standard for invasive disease; no routine surveillance in asymptomatic solid-organ recipients.	No established antiviral prophylaxis; prevention relies on minimising unnecessary immunosuppression and infection control.	Mainstay is supportive care and reduction of immunosuppression; cidofovir (with probenecid and renal dosing) used off-label in severe disease; brincidofovir under investigation; ribavirin not recommended routinely.
Parvovirus B19	Solid organ transplantation with significant immunosuppression; anaemia or cytopenias in the	Diagnosis by PCR for parvovirus B19 DNA in blood or marrow; IgM/IgG serology adjunctive; bone	No vaccine or routine prophylaxis; focus on avoiding excessive	Reduction of immunosuppression plus IVIG (commonly 400 mg/kg/day to cumulative ≈2

Virus	Key risk factors	Suggested screening & frequency	Prophylaxis / prevention	First-line treatment
	post-transplant setting.	marrow biopsy if PCR/serology negative but suspicion remains; no routine screening recommended.	immunosuppression and monitoring at-risk patients with anaemia.	g/kg, repeatable with relapse); monitor haemoglobin and repeat PCR if anaemia recurs.
Sapovirus	Kidney transplantation with impaired mucosal immunity; faecal–oral exposure; young or frail recipients at higher risk of severe diarrhoea.	Stool multiplex real-time PCR for diagnosis in symptomatic patients; no routine screening in asymptomatic recipients.	No vaccine; general enteric infection prevention (hand hygiene, food and water safety, faecal–oral transmission precautions).	Supportive care (hydration, electrolyte management, immunosuppression review); nitazoxanide has emerging but limited case-report support in renal transplant recipients.
Mpox (monkeypox)	Immunosuppressed state (particularly HIV co-infection); high-risk sexual exposure; travel or contacts from endemic/outbreak areas; kidney transplant status itself a risk for severe disease.	Diagnosis by real-time PCR on lesion material; post-transplant screening is not protocolised and is done only on clinical suspicion; donor screening follows OPTN guidance for high-risk donors.	Pre- and post-exposure vaccination with non-replicating smallpox/mpox vaccine (JYNNEOS/IMVAMUNE/IMVANEX) in eligible high-risk patients; vaccinia immunoglobulin as post-exposure prophylaxis; avoid live replicating ACAM2000 in transplant recipients.	Tecovirimat is recommended first line in high-risk patients (including kidney transplant recipients), with careful monitoring of tacrolimus and renal function; cidofovir generally avoided due to nephrotoxicity; brincidofovir is a less nephrotoxic alternative used in some transplant cases, plus supportive care and immunosuppression adjustment.

Virus	Key risk factors	Suggested screening & frequency	Prophylaxis / prevention	First-line treatment
Human Pegivirus-1 (HPgV-1)	Blood-borne exposure (e.g. haemodialysis, transfusions); parenteral and sexual transmission; kidney transplantation with prior exposure.	Diagnosis by HPgV-1 RNA PCR; antibody to envelope glycoprotein indicates clearance; no routine screening in transplant programmes.	No established prophylaxis; no routine vaccination or screening policy.	No established treatment; occasional case reports describe interferon, ribavirin or immunosuppression reduction in pegivirus-associated encephalomyelitis, but these are not evidence-based standards.
Human papillomavirus (HPV)	Sexual activity; solid organ transplantation (analogous to HIV risk); high-risk HPV types (16, 18); prolonged immunosuppression.	Cervical screening with cytology and/or HPV DNA testing: every 6 months in first post-transplant year, then annually; some centres increase frequency in high-risk HPV-positive patients; anal screening in high-risk groups (e.g. receptive anal intercourse).	HPV vaccination (bi-, quadri- or nonavalent), ideally pre-transplant and before sexual debut but can be used safely post-transplant; intensified cervical/anal screening in transplant recipients.	Local lesion-directed therapy (topicals, cryotherapy, excision/ablation) for warts and pre-malignant lesions; standard oncologic management for cancers; consider immunosuppression reduction or switch to mTOR inhibitors in refractory or recurrent disease.
Hepatitis B virus (HBV)	Haemodialysis exposure; blood products; HBV-positive donor kidneys; HBsAg or anti-HBc positivity; strong immunosuppression and B-cell depleting agents.	Pre-transplant serology (HBsAg, anti-HBs, anti-HBc) in recipients; HBsAg, anti-HBc and HBV DNA NAT in donors; ongoing post-transplant monitoring of HBV markers in	HBV vaccination early in CKD course and pre-transplant; booster doses guided by anti-HBs titres; universal antiviral prophylaxis in recipients with chronic or prior HBV and those	First-line oral antivirals (entecavir, tenofovir alafenamide) started immediately post-transplant in chronic/prior HBV; duration individualised; treat reactivation

Virus	Key risk factors	Suggested screening & frequency	Prophylaxis / prevention	First-line treatment
		infected or at-risk recipients.	receiving HBsAg+ grafts, typically for at least 6–12 months or longer.	promptly and continue close hepatic and virological monitoring.
**Hepatitis C virus (HCV)**	CKD/dialysis exposure; blood products; HCV-positive donor kidneys; immunosuppression-related progression post-transplant.	Routine HCV antibody screening in all CKD, dialysis and transplant candidates with reflex HCV RNA testing if positive; 3-monthly NAT and liver function tests post-transplant in infected recipients or those with HCV-positive donor kidneys.	No vaccine; prevention relies on infection control in dialysis units and careful donor–recipient matching; pre-emptive or prophylactic DAA use around transplantation for HCV-positive donor kidneys.	Direct-acting antiviral combinations (e.g. glecaprevir/pibrentasvir, ledipasvir/sofosbuvir, sofosbuvir/velpatasvir or elbasvir/grazoprevir) recommended in CKD, dialysis and post-transplant with attention to GFR and drug–drug interactions with immunosuppressants.

Table S1. Summary table of viruses, including important risk factors, suggested screening technique and frequency, prophylactic and first-line treatments
